# Blood‐based CNS‐Specific Extracellular Vesicles Reflecting Brain pathology as Potential Biomarkers for Alzheimer's Disease and Differential Diagnosis

**DOI:** 10.1002/alz70856_103393

**Published:** 2025-12-24

**Authors:** Chen Tian

**Affiliations:** ^1^ The First Affiliated Hospital Zhejiang University School of Medicine, Hangzhou, Zhejiang, China

## Abstract

**Background:**

Alzheimer's disease (AD) and other dementias present significant diagnostic challenges, particularly in early stages. Current diagnostic methods, such as cerebrospinal fluid (CSF) analysis and positron emission tomography (PET), are invasive, costly, and inaccessible for many patients. Extracellular vesicles (EVs) in blood, particularly those originating from brain regions like the hippocampus and cortex—areas affected early in AD—offer a promising, minimally invasive alternative for liquid biopsy. This study addresses the critical need for precise, accessible biomarkers to improve early detection and differential diagnosis of AD.

**Method:**

This multicenter study involved 523 participants, including 132 AD patients, 93 non‐AD dementia (NAD) patients, and 85 healthy controls (HCs). AD patients were confirmed through CSF and PET profiles, while NAD patients exhibited dementia symptoms without AD‐specific molecular or PET evidence. Human brain tissue samples were obtained from the National Human Brain Bank, Zhejiang University. Mouse cortical and hippocampal neuron cultures were prepared, and EVs were enriched for analysis. Mass spectrometry identified region‐specific biomarkers in EVs, which were further validated in human plasma using advanced techniques, including immunohistochemistry, electron cryo‐microscopy, nanoparticle tracking analysis, western blot, and NanoFCM Flow NanoAnalyzer.

**Result:**

We identified neurogenic EVs carrying phosphorylated tau (pTau217) as promising AD biomarkers, with significant differences in count and size distribution in plasma. Mass spectrometry of mouse cortical and hippocampal neuron EVs revealed GABRD and GPR162 as neuron‐specific markers, which were subsequently validated in human plasma using flow cytometry. These CNS EV‐tagged proteins, particularly pTau217, demonstrated high sensitivity and specificity in differentiating AD from HCs and other non‐AD dementias. The nanoflow cytometry method provided a reliable, rapid, and stable platform for plasma EV detection, reflecting CNS pathology changes through peripheral blood examination.

**Conclusion:**

Our findings demonstrate the potential of plasma neurogenic EVs carrying Aβ and pTau as highly effective biomarkers for AD diagnosis and differential diagnosis. This innovative approach holds significant promise for clinical practice, enabling earlier intervention, targeted therapy development, and improved disease management.

